# DMH1, a Novel BMP Small Molecule Inhibitor, Increases Cardiomyocyte Progenitors and Promotes Cardiac Differentiation in Mouse Embryonic Stem Cells

**DOI:** 10.1371/journal.pone.0041627

**Published:** 2012-07-27

**Authors:** Ada Ao, Jijun Hao, Corey R. Hopkins, Charles C. Hong

**Affiliations:** 1 Division of Cardiovascular Medicine, Department of Medicine, Vanderbilt University School of Medicine, Nashville, Tennessee, United States of America; 2 Vanderbilt Institute of Chemical Biology, Department of Pharmacology, Center for Neuroscience Drug Discovery, Vanderbilt University School of Medicine, Nashville, Tennessee, United States of America; 3 Vanderbilt Institute of Chemical Biology, Department of Pharmacology, Vanderbilt University School of Medicine, Nashville, Tennessee, United States of America; 4 Department of Cell and Developmental Biology, Vanderbilt University School of Medicine, Nashville, Tennessee, United States of America; 5 Research Medicine, Veterans Affairs Tennessee Valley Healthcare System, Nashville, Tennessee, United States of America; University of Otago, New Zealand

## Abstract

The possibility of using cell-based therapeutics to treat cardiac failure has generated significant interest since the initial introduction of stem cell-based technologies. However, the methods to quickly and robustly direct stem cell differentiation towards cardiac cell types have been limited by a reliance on recombinant growth factors to provide necessary biological cues. We report here the use of dorsomorphin homologue 1 (DMH1), a second-generation small molecule BMP inhibitor based on dorsomorphin, to efficiently induce beating cardiomyocyte formation in mouse embryonic stem cells (ESCs) and to specifically upregulate canonical transcriptional markers associated with cardiac development. DMH1 differs significantly from its predecessor by its ability to enrich for pro-cardiac progenitor cells that respond to late-stage Wnt inhibition using XAV939 and produce secondary beating cardiomyocytes. Our study demonstrates the utility of small molecules to complement existing in vitro cardiac differentiation protocols and highlights the role of transient BMP inhibition in cardiomyogenesis.

## Introduction

The irreversible loss of cardiomyocytes following myocardial infarction causes the clinical features of heart failure, marked by regional contractile dysfunction manifesting mainly in the ventricular chamber. The advent of stem cell biology and regenerative medicine offer enticing prospects for manufacturing specific cell types from pluripotent cells, which may be used to replace or repair damaged tissues. This is preferable to traditional organ transplants because donor availability and compatibility constitutes a significant barrier. However, the expected cell-based therapeutics have not matured as the directed differentiation process is inefficient, resulting in a heterogeneous cell population that risks further complications if implanted into patients. There are also technical hurdles against the large-scale production of clinical grade products because current protocols rely on the use of animal-derived growth factors, which may introduce batch-to-batch variability that constitutes additional safety concerns for humans [1]–[3]. Therefore, there is an urgent need to develop tools for directed differentiation that are both xeno-free and have robust biological effects.

Insights from developmental biology studies have uncovered key molecular pathways that guide mammalian cardiac differentiation. The process of cardiomyocyte development from mesoderm progenitors requires coordinated changes in BMP signaling along with other mitogenic pathways including Activin, FGF, and Wnt signaling [4]–[8]. Previous studies have shown that the simple presence of BMP ligands is insufficient to initiate cardiac differentiation [6], [9], and BMP signaling in mesoderm is sequentially and locally controlled by antagonists secreted from the surrounding ectoderm and endoderm during cardiac morphogenesis [10]–[12]. Recent studies also suggested that the timing and the duration of BMP signaling in pluripotent cells may influence atrial and ventricular lineage commitment of multipotent cardiac progenitors [13]–[15]. An overall picture emerges in which early BMP signaling modulation is not only necessary to specify the cardiac progenitor pool, but also to temporally regulate cardiac chamber development.

Small molecules have emerged as an adaptable tool that take advantage of insights borrowed from developmental biology. They have been used for directing differentiation and have demonstrated their advantages over the use of recombinant proteins in many aspects of regenerative medicine [16]–[18]. Our previous study, which described the use of dorsomorphin (DM) to mimic the function of endogenous BMP inhibitor Noggin for directing cardiomyocyte formation in mouse embryonic stem cells, demonstrated that the timely application of a single chemical can be a viable strategy for directed cardiac differentiation [19]. However, DM was later shown to target not only Smad-dependent signaling, but it also targeted AMP-kinase (AMPK) and receptor tyrosine kinases for PDGF and VEGF signaling [20]–[22]. Hao et al. [19] speculated that non-BMP signaling may have induced cardiomyogenesis and may also account for the delayed or limited induction of early cardiac differentiation markers in that study. Therefore, this study proposes to investigate the cardiomyogenic molecular profile using a second-generation small molecule BMP inhibitor, dorsomorphin homologue 1 (DMH1), which was synthesized and characterized in a large-scale in vivo structure-activity relationship (SAR) study [21]. DMH1 was shown to be a far more selective inhibitor of BMP Type 1 receptors than DM and LDN-193189 [23], [24] and did not possess inhibitory activity for p38 MAPK phosphorylation, Activin A-induced Smad2 phosphorylation, or VEGF-induced Flk1 phosphorylation [21]. We report here a detailed comparison of DM and DMH1 in the context of cardiomyogenic induction in mouse embryonic stem cells. In doing so, we uncovered additional advantages presented by DMH1 and its ability to affect early cell fate commitment that can contribute to late-stage cardiomyogenesis.

**Figure 1:**
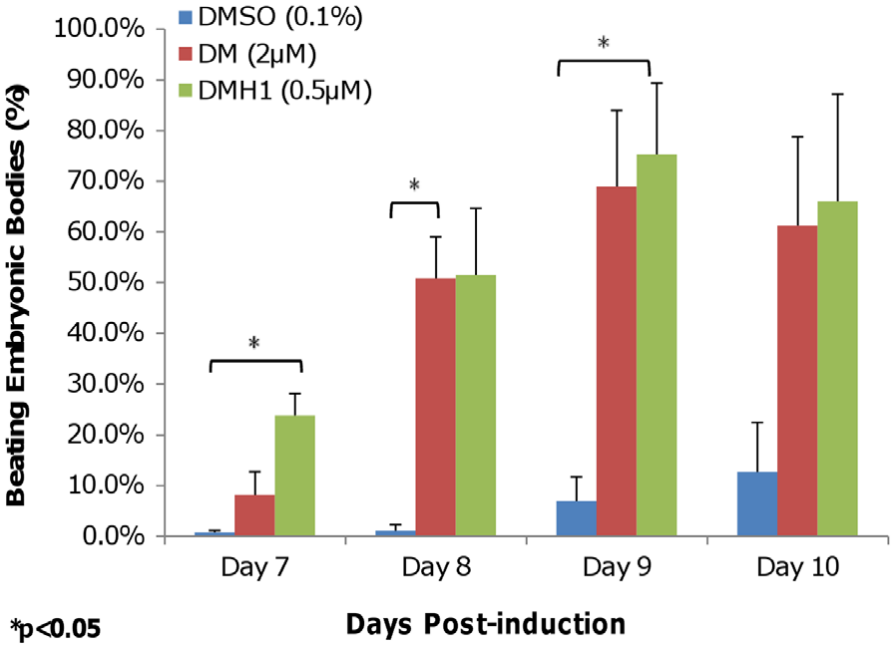
DMH1 induces cardiomyogenesis in mouse ES cells. The data show both DM and DMH1 treatment can induce beating EB formation beginning on Day 7 and that the percentage of beating EBs increases over time. Both compounds have comparable levels of induction efficiency that are significantly higher than DMSO control. Results are presented as the average of three independent experiments. Error bars denote the S.E.M. (standard error of the mean). P-value is calculated using two-tailed Student’s t-test. DMSO is the vehicle control. DM is dorsomorphin treatment. DMH1 is dorsomorphin homologue 1.

## Materials and Methods

### Mouse Embryonic Cell Lines and Maintenance

CGR8 mouse embryonic cells were kindly provided by Antonis Hatzopoulos (Vanderbilt University), which were first described in [25]. The cells were maintained on 0.2% gelatin-coated dishes in maintenance media composed of GMEM (Sigma) supplemented with 10% HI-FBS (Gibco), 2 mM L-glutamine (Sigma), 0.5 M 2-Mercaptoethanol (Sigma), and 200 U/mL mLIF (Millipore).

**Figure 2:**
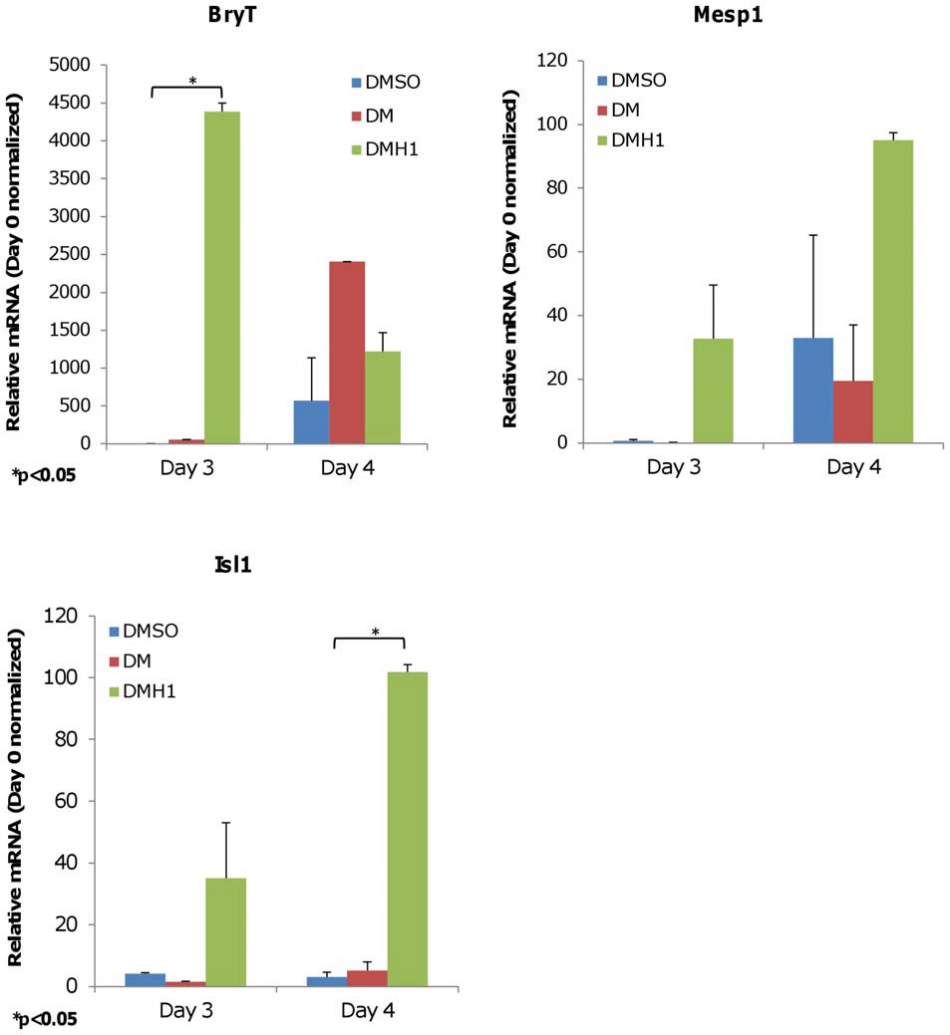
DMH1-treated cells show upregulated expression of mesoderm markers. CGR8 cells were induced in differentiation media containing various compounds to form EBs using the hanging-drop method for 48 hours. The data show DMH1 induces upregulation of transcription factors for pre-mesoderm progenitors (BryT), mesoderm commitment (Mesp1), and second heart field (Isl1) compared with DMSO control; while DM treatment induces only limited or delayed induction of the same markers. Results are presented as the average of three independent experiments. Expression levels shown are normalized to Day 0 expression levels. Error bars denote the S.E.M. (standard error of the mean). P-value is calculated using two-tailed Student’s t-test. DMSO is the vehicle control. DM is dorsomorphin treatment. DMH1 is dorsomorphin homologue 1.

**Figure 3:**
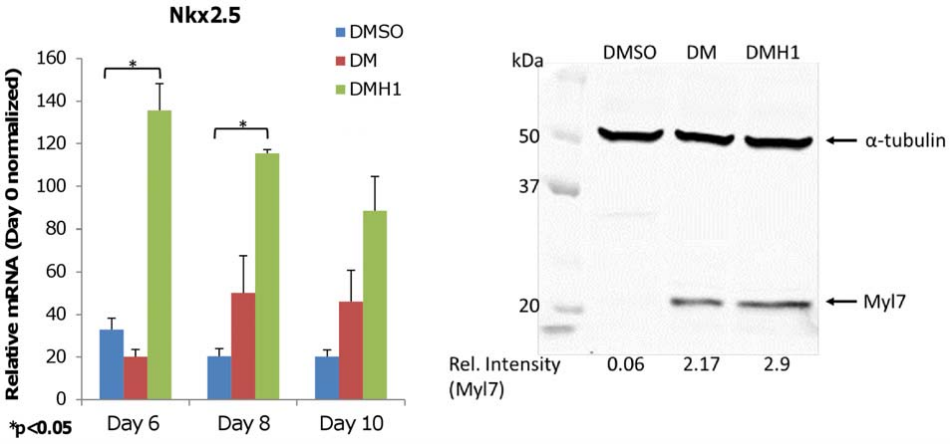
DMH1-treated cells showed increased cardiomyocyte lineage commitment. (Left) DMH1 induces significant upregulation of transcription factor for cardiomyocyte commitment (Nkx2.5) compared with DMSO control. DM also induces Nkx2.5 expression, but the induction is less robust than DMH1. Results are presented as the average of three independent experiments. Expression levels shown are normalized to Day 0 expression levels. Error bars denote the S.E.M. (standard error of the mean). P-value is calculated using two-tailed Student’s t-test. (Right) Western blot showing increased Myl7 expression (∼19 kDa), a structural protein specific to cardiomyocytes, in CGR8 cells 8 days after DM or DMH1 induction. No Myl7 expression was observed after DMSO treatment. Alpha-tubulin (∼50 kDa) is shown as loading control. The relative intensities of Myl7 bands are normalized to alpha-tubulin and are shown at the bottom.

Feeder-dependent R1 and BryT-GFP cells were kind gifts from Eric Adler (Oregon Health Science Center) and were previously described [26]. The cells were maintained on mitomycin C-inactivated SNL cells (gift from Kevin Ess at Vanderbilt), which were first described in [27]. They were plated onto 0.1% gelatin-coated dishes, in High Glucose DMEM (Gibco #11960) supplemented with 15% HI-FBS (Gibco), 2 mM L-glutamine (Sigma), 1X nonessential amino acids (Sigma), 1% Pen-Strep (Gibco), 0.05 mM 2-mercaptoethanol (Sigma), 1 mM sodium pyruvate (Sigma), and 200 U/mL mLIF (Millipore). The media was changed daily for all mouse embryonic cell lines prior to differentiation induction.

**Figure 4:**
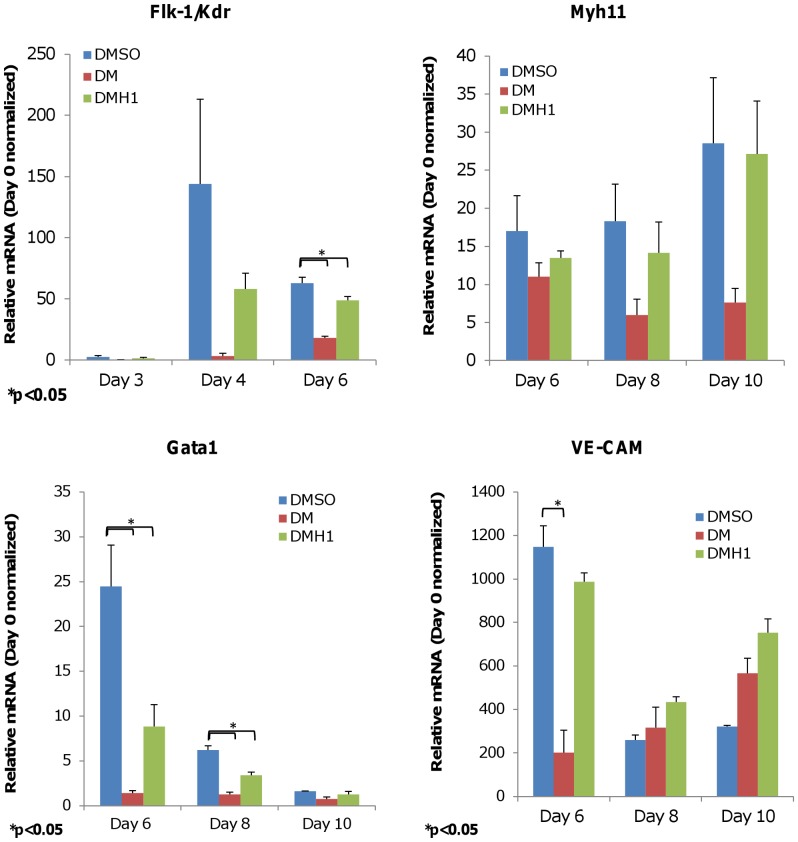
DMH1 treatment does not specifically induce other mesoderm-associated lineage markers. CGR8 induction and gene expression analysis were performed as previously described. The data show DM or DMH1 treatment does not specifically induce hematopoietic markers Gata1 and Flk-1/Kdr, or smooth muscle marker Myh11 when compared with DMSO control. Both compounds show a modest induction of vascular endothelial marker VE-CAM but the differences are not statistically significant. Results are presented as the average of three independent experiments. Expression levels shown are normalized to Day 0 expression levels. Error bars denote the S.E.M. DMSO is the vehicle control. DM is dorsomorphin treatment. DMH1 is dorsomorphin homologue 1.

### Small Molecules

The synthesis and characterization of dorsomorphin (DM), dorsomorphin homologue 1 (DMH1), dorsomorphin homologue 4 (DMH4), and Wnt inhibitor XAV939 were described previously [21], [28], [29]. All experiments were performed using 2 µM DM, 0.5 µM DMH1, 2 µM DMH4, or 1 µM XAV939 diluted in DMSO.

**Figure 5:**
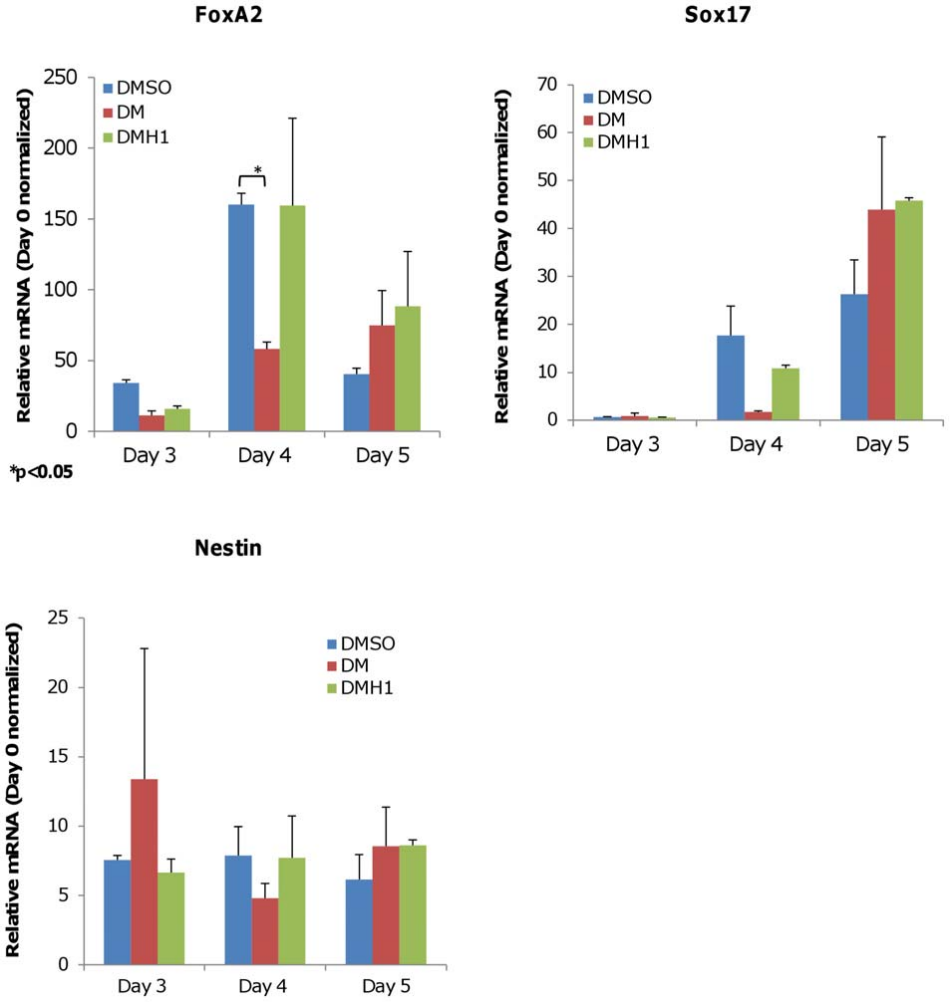
DMH1 treatment does not specifically induce expression of endoderm or ectoderm markers. DMH1 treatment does not have a statistically significant effect on definitive endoderm markers FoxA2 and Sox17, or ectoderm progenitor marker Nestin. The data show DM treatment results in a statistically significant reduction in FoxA2 expression on Day 4, and a general delay in Sox17 expression. There is no significant increase in the expression of Nestin after DM treatment. Results are presented as the average of three independent experiments. Expression levels shown are normalized to Day 0 expression levels. Error bars denote the S.E.M. P-value is calculated using two-tailed Student’s t-test. DMSO is the vehicle control. DM is dorsomorphin treatment. DMH1 is dorsomorphin homologue 1.

### Cardiac Differentiation

For CGR8 differentiation, the media was composed of IMDM (Gibco) supplemented with 20% HI-FBS (Gibco), 2 mM L-glutamine (Sigma), 1X nonessential amino acids (Sigma), and 0.1 mM 2-mercaptoethanol (Sigma).

**Figure 6:**
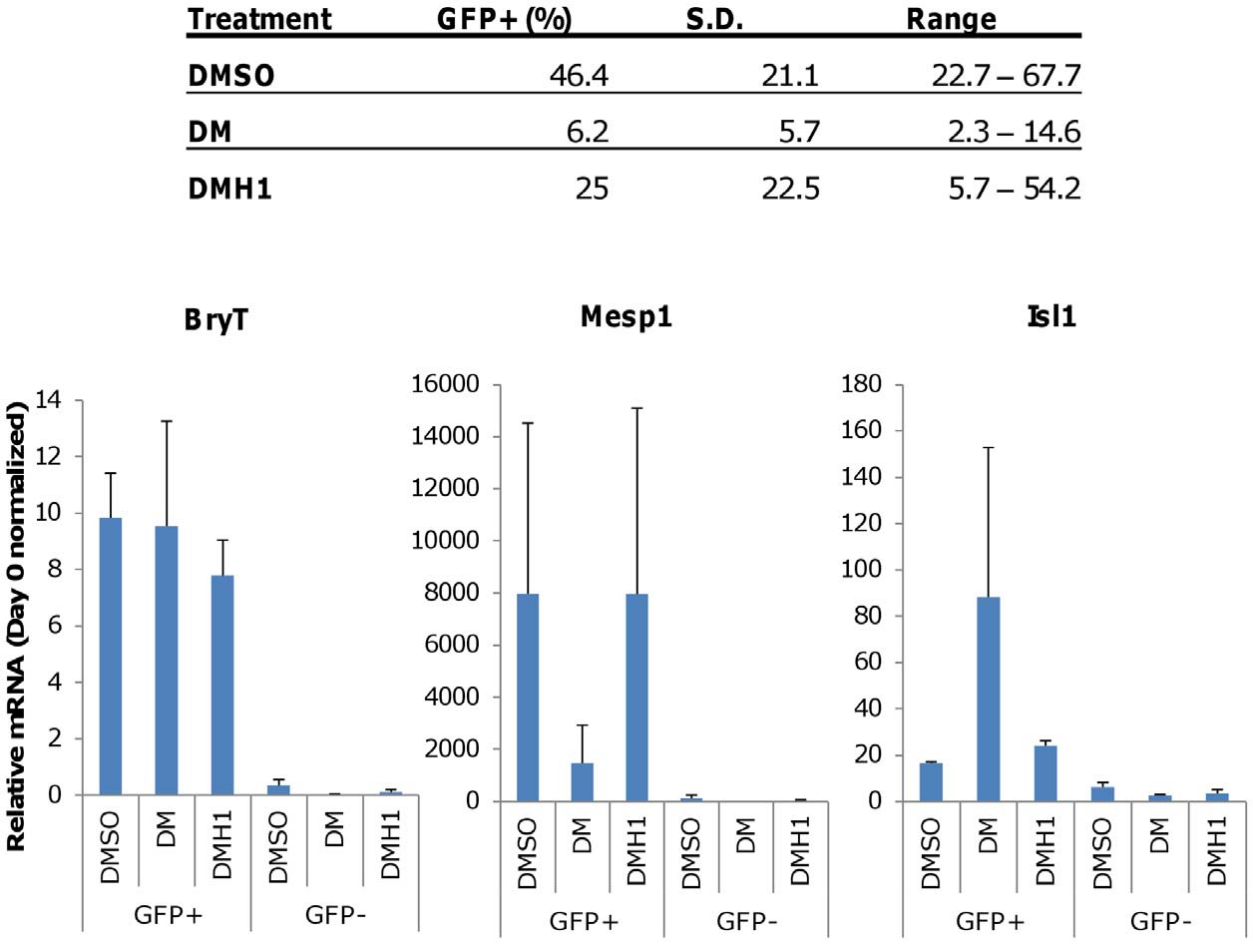
FACS analysis confirms BryT induction by DMH1 and limited BryT induction by DM. Mouse embryonic stem cells containing a recombinant BryT-GFP reporter were induced as previously described and analyzed using FACS after 4 days of treatment with various compounds. (Top) The percentages of GFP+ cells calculated from at least 10,000 parental cells are shown. The range shows the variability of total GFP+ cells for the experiments. The data show DM treatment yields fewer GFP+ cells than DMH1-treated cells. At least two independent experiments are presented as the mean. SD is standard deviation. (Bottom) BryT expression levels in the GFP+ fractions are confirmed using rt-PCR. GFP+ cells also show increased Mesp1 and Isl1 expression, which indicates a transition to mesoderm and cardiac lineage commitment. Rt-PCR results are presented as the average of three independent experiments. Expression levels shown are normalized to Day 0 expression levels. Error bars denote the S.E.M. DMSO is the vehicle control. DM is dorsomorphin treatment. DMH1 is dorsomorphin homologue 1.

For R1 and BryT-GFP differentiation, the cells were passaged at least twice onto 0.2% gelatin-coated dishes to remove feeder cells, and embryonic culture media as described above supplemented with an additional 1000 U per mL mLIF. To initiate differentiation, the cells were dissociated with 0.5% trypsin-EDTA and resuspended in differentiation media composed of IMDM (Gibco) supplemented with 20% HI-FBS (Gibco), 2 mM L-glutamine (Sigma), 1X nonessential amino acids (Sigma), 0.1 mM 2-mercaptoethanol (Sigma), and 1 mM sodium pyruvate (Gibco).

**Figure 7:**
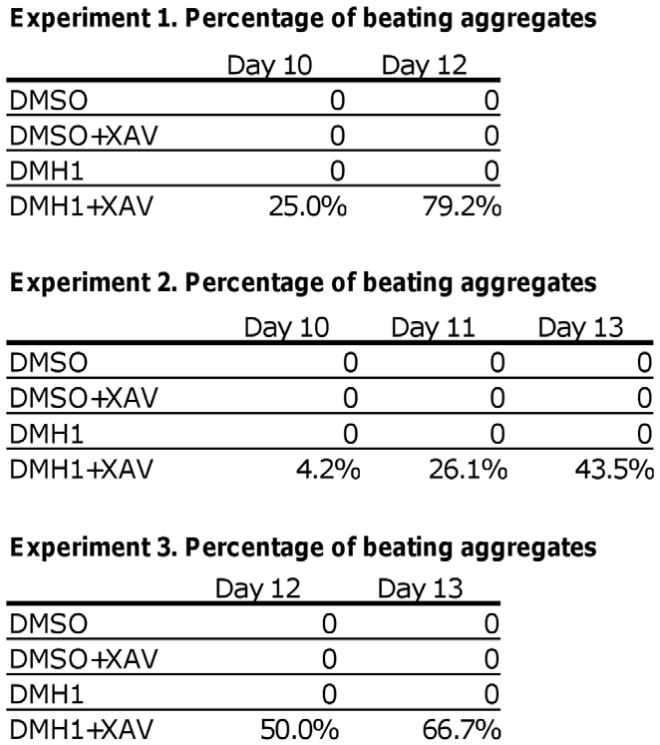
Low BryT-expressing fraction produces beating EB after Wnt inhibition. Each table is data from a single experiment showing the percentage of beating aggregates that formed after GFP– fractions were treated with either DMSO control or 1 µM XAV939 to inhibit Wnt signaling. The DMH1-treated GFP– fractions typically form beating aggregates 6 to 8 days after XAV939 addition (Day 10 to Day 12 after initial induction). The percentages increase over time and the efficiency of beating aggregate formation varies with each FACS. The percentages were calculated from the number of beating aggregates and the number of total aggregates for each condition.

Differentiation was initiated using the hanging-drop method. Briefly, mouse embryonic cells were trypsinized and resuspended in differentiation media at 25,000 cells per mL. Next, 20 µL drops were placed onto an inverted 15-cm petri dish lid under humidified conditions, and cells were allowed to aggregate in the droplet for 1–2 days to form embryonic bodies (EBs). The media was changed 2 days after induction by washing the EBs with fresh media, and then continuing suspension culture in petri dishes. The EBs were transferred onto 0.2% gelatin-coated culture vessels 4 days after induction to allow attachment. The media was changed every 2 days thereafter.

### RNA Extraction and cDNA Synthesis

Cell samples were homogenized using QIAshredder (Qiagen) according to manufacturer’s instructions. RNA extraction was performed using RNeasy Mini Kit (Qiagen) according to manufacturer’s instructions. Extracted total RNA (1–2 µL) was immediately used for cDNA synthesis. cDNA synthesis was performed using SuperScript III (Invitrogen) according to manufacturer’s protocol with 50 ng random hexamer primers in a 20 µL reaction. The resulting cDNA was diluted 1∶5 in ddH2O prior to real-time PCR.

### Taqman Real-time PCR (rt-PCR)

One microliter of diluted cDNA was used in each 20 µL reaction, along with 1 µL Taqman probe (Applied Biosystems, ABI), and 10 µL 2X Taqman Universal PCR Master Mix (ABI). The Taqman probed used to evaluate gene expression were as follows: mGAPDH (Mm99999915_g1), mGATA1 (Mm00484678_m1), mMesp1 (Mm00801883_g1), mMyh11 (Mm00443013_m1), mNkx2.5 (Mm00657783_m1), mBryT (Mm00436877_m1), mTbx18 (Mm00470177_m1), mIsl1 (Mm00627860_m1), mKdr (Mm00440099_m1), mFoxA2 (Mm00839704_mH), mSox17x (Mm00488363_m1), and mNestin (Mm00450205_m1). Each cDNA sample was analyzed in triplicate in a 384-well plate. The expression levels shown are normalized to Day 0 and represents mRNA increases that are above those on Day 0. The results were averaged from three independent experiments and displayed as mean ± standard error of the mean (S.E.M.). The p-value was calculated using two-tailed Student’s t-test and compares DMSO control treatments vs. DM or DMH1; p-values of <0.05 are considered to be significant. Treatments that induced greater than 2-fold relative expression changes when compared with DMSO are considered to be specific.

### FACS

After 4 days of cardiac induction, EBs formed from BryT-GFP cells as hanging drops were collected in 15-mL conical tubes and allowed to settle by gravity. The supernatant was removed, and the EBs were washed 1X in PBS, and allowed to settle again by gravity. The PBS wash was removed, and the collected EBs were resuspended in 1 mL 0.05% trypsin-EDTA and incubated at 37°C for 3–5 minutes. Four milliliters of differentiation media was added to quench the digestion, and then the EBs were manually dissociated by repeat pipetting using a 5-mL pipette. The dissociated cells were centrifuged and the supernatant was removed. The cell pellet was washed twice in 1X Hank’s Balanced Salt Solution (1X HBSS, Sigma) before resuspension in 250–300 µL of cold cell sorting buffer (1X HBSS with 5% HI-FBS) and kept on ice until sorting. R1 cells were cultured and induced in parallel with BryT-GFP cells and served as GFP-negative gating control.

Sorted cells are collected in differentiation media. The sorted samples are centrifuged and resuspended to a final cell density of 100,000 cells per mL in differentiation media. Small molecules or vehicle were added to the cell suspension, as appropriate. The samples were then replated at 100 µL per well in U-shaped, 96-well low attachment plates (Corning), and allowed to reaggregate for 2 days. The media was changed every 2 days thereafter, samples for RNA extraction were collect, and beating aggregates were manually scored.

### Beating Embryonic Bodies (EB) Quantification

The protocol for beating EB quantification was described previously [30]. Briefly, mouse embryonic cells were induced under cardiogenic conditions, and resuspended at either 5,000 cells per mL for CGR8 induction, or 100,000 cells per mL for FACS-sorted cells. The cell suspensions were plated at 100 µL per well in U-shaped, 96-well low attachment plates (Corning), and cultured for 2 days so that each well contains a single aggregated EB. The media was changed every 2 days thereafter. The wells were observed from Day 7 to Day 13 after initial induction. Wells were scored manually for beating EB activity and the percentage was calculated for each condition according to the number of wells containing beating EBs and the total number of EBs plated. The results are averaged from three independent experiments and presented as mean ± standard deviation (SD) unless otherwise noted.

### Western Blot Analysis

Induced CGR8 EBs were homogenized in CelLytic M (Sigma) according to manufacturer’s instructions. Whole cell lysate was separated on a 10% SDS-PAGE gel and transferred onto nitrocellulose membrane. The membrane was blocked in Odyssey Blocking Solution (Li-Cor Biosciences), and probed using antibodies diluted in the blocking solution. The antibodies used were myosin heavy chain 7 (Myl7, clone B-10) from Santa Cruz Biotechnologies at a dilution of 1∶100, and alpha-tubulin (Clone 11H10, Cell Signaling Technologies) at a dilution of 1∶5000. Relative intensities of the observed bands were analyzed using ImageJ software (http://rsb.info.nih.gov/ij/).

### TUNEL Assay

CGR8 cells were seeded at low density onto 1% gelatin-coated chamber glass slides for 2 days in GMEM-based maintenance media as described above. To begin the experiment, the culture media was changed to CGR8 differentiation media (IMDM-based) containing DMH1, DM, or vehicle at experimental concentrations. The cells were incubated for 1–2 days before cellular apoptosis was assayed using In Situ Cell Death Detection Kit–Fluorescein (Roche) according to manufacturer’s instructions.

## Results

### Treatment with DMH1 is Sufficient to Induce Cardiomyogenesis

To gauge the cardiogenic efficiency of DMH1, we quantify contracting embryonic bodies (EBs) by aggregating CGR8 mouse embryonic stem cells in low-attachment U-shaped 96-well plates as previously described [30] to permit scoring after chemical treatments. We rely on the beating EB assay in place of protein-based assays for cardiomyocyte identification because the presence of cardiac-specific proteins does not necessarily correlate with cardiomyocyte contractility. In contrast, the ability to contract indicates that the necessary cardiac-specific proteins are present to allow function. As shown in Figure 1, contracting EBs began to appear on Day 7 after DMH1 induction at about 20% efficiency and peaked at about 70%–75% efficiency for both DM and DMH1 induction on Days 9 to 10. Both DM-and DMH1-treated EBs showed a consistent tendency to significantly increase beating EB formation compared with DMSO vehicle control, which remained at <10% efficiency for all observed time points.

When DMH1 is compared with DM, we observed that DMH1-induced EBs began to beat a day earlier than DM (Figure 1). However, this difference is transitory as the percentage of DM-induced beating EBs approached those formed by DMH1 induction on Day 8, and the two percentages were very similar on Days 9 to 10. The earlier appearance of beating EBs in DMH1-treated samples may be an indication of the compound’s greater BMP inhibition specificity.

### DMH1 Treatment Induced a Cardiogenic Molecular Expression Profile that Differs from DM Treatment

DMH1 treatment upregulated transcription factors associated with the mesoderm lineage and cardiac differentiation when compared with vehicle control and DM treatment. DMH1 significantly induced pre-mesoderm marker *BryT* expression on Day 3 that persisted to Day 4, and mesoderm lineage marker *Mesp1* on Day 3 and Day 4 (Figure 2). DMH1 also upregulated *Isl1* expression, which is generally associated with development of the second heart field [31], [32] and neural crest cardiac progenitors [33]. During later stages of cardiac differentiation, we observed a statistically significant increase of cardiomyocyte-specific *Nkx2.5* expression in DMH1-treated samples beginning on Day 6 (Figure 3), which may account for the somewhat earlier appearance of beating EBs compared with DM-treated samples.

DMH1-induced cells displayed a markedly different expression profile than the previously described DM. DM-treated cells showed a generally delayed or limited expression of pre-mesoderm and mesoderm-specific transcription factors *BryT* and *Mesp1,* respectively (Figure 2) that were consistent with previous results [19]. Furthermore, DM did not significantly induce *Isl1* expression and showed a less robust induction of *Nkx2.5* expression (Figures 2 and 3). However, this difference in cardiomyogenic commitment disappears by Day 8 as shown by myosin light chain (Myl7) protein expression, a structural protein specific to cardiomyocytes, which showed comparable levels for both DM and DMH1 induction (Figure 3). The resulting expression profiles suggested that DMH1-induced cardiomyogenesis involves a canonical, step-wise pathway that requires known pre-mesoderm and mesoderm-specific transcription factors to regulate cardiomyocyte commitment. The profiles also confirmed previously published data that DM induced limited or delayed expression of mesoderm-associated factors, and suggested that DM-induced cardiomyocytes developed from a non-canonical pathway that remains unknown.

### DMH1 does not Specifically Induce Other Mesoderm-associated Lineage Markers

*BryT*-committed cells are known to be multipotent cells capable of producing hematopoietic progenitors [34] and other mesoderm-associated lineages such as smooth muscle cells and endocardial cells [35]. Therefore, we further probed the DMH1-induced gene expression profile for signs of hematopoiesis and non-cardiomyocyte development. In Figure 4, transcriptional profiling of DMH1-induced EBs indicated that, similar to DM [19], DMH1 did not specifically induced the expression of multipotent progenitor marker *Kdr/Flk-1* or hematopoiesis (*Gata1*) compared with DMSO induction, which stimulates spontaneous and non-specific differentiation. Similarly, DMH1 treatment did not induced specific *Myh11* expression, a marker for smooth muscle cells, relative to non-specific DMSO induction as the two expression levels are comparable.

However, DMH1 supported a modest 2.3-fold increase in the expression of endocardial marker *VE-CAM* on Day 10 (Figure 4) when compared with DMSO, but it is unclear if this modest increase contributes to further endocardial lineage development.

Overall, DMH1 treatment did not specifically increase *Kdr/Flk-1*, *Gata1*, or *Myh11* expression levels relative to non-specific DMSO induction. This is unlike DM treatment, which showed a notable decrease in the expression of those three genes relative to DMSO. This pattern suggested that DM may specifically repress their expression and is consistent with previously published results [19].

### DMH1 does not Specifically Induce Endoderm or Ectoderm Markers

DMH1-induced EBs were also examined for endoderm or ectoderm commitment to confirm mesoderm induction specificity. As shown in Figure 5, DMH1 did not specifically upregulate endoderm markers *Sox17* or *FoxA2* expression in a statistically significant manner when compared with DMSO from Day 3 to Day 6. In addition, the increases observed for DMH1 treatments were less than 2-fold, which is within the margin of error for rt-PCR analysis and therefore were not considered a meaningful increase. There was also sufficient variability between each independent experiment to indicate that the observed *Sox17* or *FoxA2* expression level may be either randomly, or minimally, induced by DMH1. The ectoderm marker *Nestin* was also minimally affected during the early stages of DMH1-induced differentiation. We did not observe statically significant *Nestin* induction by DMH1 relative to DMSO. We concluded that the influence of DMH1 on directed differentiation during the earliest stages of induction is restricted to the mesoderm linage.

DM treatment appeared to delay the expression of endoderm markers Sox17 and FoxA2 at the time points examined (Figure 5). But like DMH1, the observed increases in expression appeared to be non-specific and are not statistically significant changes. The expression level of *Nestin* was also not significantly affected by DM.

### DMH1 Treatment Enriched for Pro-cardiac Progenitors that Responded to Wnt Inhibition and Progressed to Form Secondary Cardiomyocytes

The marked difference in the pro-cardiac gene expression profiles between DM and DMH1 prompted us to further investigate their respective developmental potential at the pre-mesoderm and early mesoderm stages following treatment. We used a mouse embryonic stem cell line containing a recombinant *BryT*-GFP reporter that was described previously [26], [36] to isolate *BryT*-GFP positive (GFP+) and negative (GFP–) cell fractions 4 days after induction. The Day 4 time point was chosen to obtain the maximum number of GFP+ cells from all treatments for further experiments, as Day 3 FACS resulted in too few cells for additional studies. Figure 6 shows the average percentage of GFP+ cells for each treatment. The data showed that DMSO treatment yielded a greater percentage of GFP+ cells than DMH1 treatment. This observation may be due to differences in temporal *BryT* regulation by the two treatments (Figure 2). Since the cells were sorted on Day 4, the GFP signal in DMH1-treated samples may be residual signal from Day 3, whereas the signal from DMSO treatment may be nascent. There is also a large degree of experimental variation and each cell sorting yields a unique percentage of *BryT*-GFP positive cells. *BryT* expression was confirmed in the GFP+ fractions using rt-PCR and the data showed that there is no significant *BryT* expression leakage in the GFP–fractions (Figure 6). The GFP+ fractions also retained cells with upregulated *Mesp1* and *Isl1* expression levels. Their expression levels were highly variable, which suggested the GFP+ cells were transitioning to the mesoderm lineage at the time of sorting. Both *Mesp1* and *Isl1* expression were at barely detectable levels in the GFP– fractions and indicated they had not undergone mesoderm commitment.

We decided to track the cardiac development potential of all sorted cell fractions as an indirect measurement of pro-cardiomyogenic progenitor enrichment following DM and DMH1 treatment. We hypothesized that the DM-or DMH1-treated GFP+ fractions will generate beating cardiomyocytes, whereas the DMSO-treated fractions and the DM/DMH1-treated GFP– fractions will not because they lack pro-cardiac cells. Wnt signaling has been shown to have a biphasic role during cardiac development, and its inhibition is required for cardiomyocyte specification after mesoderm commitment [8], [37]–[39]. Therefore, we reasoned that the administration of small molecule Wnt inhibitor XAV939 [29] to sorted cells would mimic the biological and temporal cues for cardiomyocyte specification. The sorted fractions from DMSO-, DM-, and DMH1-treated cells were reaggregated in the presence of 1 µM XAV939 for 48 hours. All GFP+ fractions were unresponsive to XAV939 treatment and did not form beating aggregates in suspension, or formed beating colonies when the GFP+ fractions were supported by OP-9 feeder cell co-culture (data not shown). We also attempted to induced beating colony formation from all treated GFP+ fractions by culturing in differentiation media enriched with recombinant growth factors as previously described [35], [40], but was unsuccessful (data not shown). Cardiac troponin-T staining performed on all GFP+ fractions under these culture conditions were also negative (data not shown), which indicated cardiomyocytes were not produced. We hypothesized that treated GFP+ cells required intercellular signals supplied by GFP– cells that our culture conditions cannot reproduce.

Surprisingly, the DMH1-treated GFP– fractions responded to XAV939 treatment and formed beating aggregates 6 to 9 days after sorting and Wnt inhibition (Day 10 to 13 after DMH1 treatment). Vehicle-treated cell fractions did not produce beating aggregates in the presence or absence of XAV939. DM-treated fractions can also form beating aggregates, but their occurrences were few and sporadic, which may reflect the suboptimal specificity of the compound (data not shown). The percentage and the timing for the appearance of beating aggregates varied with each cell sorting (Figure 7), and their gene expression profiles reflected similar heterogeneity (Figures S1, S2, S3). Gene expression profiling revealed that XAV939 induced a second wave of pro-cardiomyocyte gene expression as shown by increased *Mesp1* and *Isl1* expression 2 to 4 days after Wnt inhibition (Day 6 to 8 after DMH1 treatment), and by increased *Nkx2.5* expression 4 to 6 days after XAV939 addition (Day 8 to 10 after DMH1 treatment) (Figures S1, S2, S3). The consistent appearance of beating aggregates in the DMH1-treated GFP– fractions after Wnt inhibition suggested that the fraction is enriched in pro-cardiomyogenic progenitors that can respond to relevant cues and produce additional cardiomyocytes.

## Discussion

Novel small molecules are increasingly engaged for the advancement of cell-based therapeutics. Compounds that can modulate specific developmental signaling pathways and promote consistent phenotypes are promising tools for analyzing the sequential steps in cell fate commitment. We report here the use of DMH1, a second-generation BMP inhibitor, to induce cardiomyocyte formation in mouse embryonic stem cells. We also showed that DMH1 enrich for pro-cardiomyogenic progenitors that can respond to late-stage developmental signals and undergo a second phase of cardiomyogenesis.

Our previous study showed that transient BMP signaling modulation can be achieved using a single synthetic molecule, DM, and a brief period of inhibition is sufficient to induce cardiomyogenesis [19]. However, DM was subsequently found to inhibit non-BMP pathways [20]–[22], which may contribute to the unusual cardiomyogenic molecular profile described in the earlier study. Therefore, a reexamination and a comparison of the molecular profiles induced by our unique compounds are warranted. DMH1 is similar to DM in the context of cardiomyocyte induction efficiency. Both compounds can induce at least a 5-fold increase in beating EB formation, showed remarkable specificity for cardiomyocyte-related gene upregulation, and did not specifically increase non-mesoderm and non-cardiomyocyte gene expression. The two compounds differ significantly in their ability to modulate pre-mesoderm (*BryT*) and early mesoderm (*Mesp1*) gene expression as shown by rt-PCR and by the behavior of early progenitor cells. We speculate that the higher BMP inhibition specificity of DMH1 may be the contributing factor to the upregulation of known cardiogenic markers in our model. We do not believe our observations are the result of selective cytotoxicity from our compounds. TUNEL assays showed that DM treatment is capable of inducing apoptosis 24 hours after initial treatment, but it was observed in only a small portion of the cell population (Figure S4). DMH1 did not appear to initiate apoptosis at 24 to 48 hours (Figures S4–S5). Therefore, it is unlikely that DMH1 treatment is selectively enriching for cardiogenic cells by inducing apoptosis based on the growth kinetics of specific cell types.

Our observations are consistent with other studies that described the temporal modulation of BMP and Wnt signaling during cardiomyogenesis [8], [37], [39], [41]. We confirmed that transient BMP inhibition is sufficient to initiate cardiomyogenesis and small molecules can serve as synthetic tools for this function [19], [41]. We also showed that Wnt inhibition at a later stage can initiate cardiomyogenesis in our low *BryT*-expressing cell fractions [8], [37], [38]. This effect is reminiscent of the second heart field, in which a second wave of cardiovascular development occurs that contributes to the formation of the outflow tract and the right ventricle, and Wnt signaling modulation has been shown to regulate its development [8]. Our model cannot determine the specific progenitor population Wnt inhibition is targeting. The beating EBs may have derived from residual mesoderm-committed progenitors in the GFP– fractions, or they may be multipotent cells that initiated mesoderm commitment and cardiomyocyte differentiation after Wnt inhibition. Based on the upregulation of *Mesp1* and *Isl1* expression 2 to 4 days after XAV939 treatment (Figures S1, S2, S3), we suspect that the secondary cardiomyocytes were derived from pro-cardiac multipotent progenitors in the GFP– fractions that were directed by late-stage Wnt inhibition towards cardiac lineage commitment. Our results are consistent with the sequential nature of directed differentiation and that specific signaling modulation during early development can produce a more restricted progenitor lineage that favors particular cell types.

Our data suggests that selective inhibition of BMP Type 1 receptors during early development is critical for increasing the proportion of cardiomyogenic progenitor cells and maximizing the overall cardiomyocyte induction potential. When the gene expression profiles of DM-or DMH1-treated cells were compared with those treated with DMH4, which is a selective VEGF-signaling inhibitor with no BMP inhibition activity [21], we observed no cardiomyogenic gene upregulation by DMH4 (Figure S6). Instead, DMH4 treatment appeared to favor vascular development as shown by the upregulation of smooth muscle marker Myh11 and endocardial marker VE-CAM (Figure S6). Furthermore, other DM homologues that are structurally similar to DMH1 and are also selective inhibitors of BMP Type 1 receptors can initiate beating EB formation ([21], unpublished data). This observation suggests that modulating only a small subset of BMP Type 1 receptors is sufficient to initiate cardiomyogenesis.

In summary, our study presents evidence that our second-generation BMP inhibitor DMH1 has more specific cardiomyogenic properties than dorsomorphin in the context of in vitro directed differentiation. DMH1 has a greater effect on early cardiac development than dorsomorphin and may enrich for pro-cardiac multipotent progenitors that can respond to late-stage Wnt inhibition and initiate a second phase of cardiomyogenic commitment. We also postulate that modulation of a small subset of BMP Type 1 receptors is central for this phenomenon. We believe DMH1 can be part of a multi-step strategy to increase cardiomyocyte induction from pluripotent cells.

## Supporting Information

Figure S1 **Gene expression analysis of individual experiments for mesoderm and cardiogenic markers for experiment No.1.** Induction of mesoderm and cardiac specific markers in the GFP– fractions after Wnt inhibition were analyzed using rt-PCR and shown as individual experiments in each figure. XAV939 (1 µM) upregulated Mesp1 expression 2 to 4 days after treatment in the sample initially induced using DMH1. The DMH1-treated fraction shows increased Isl1 expression 4 days after XAV939 treatment (Day 8 post-DMH1 induction). The expression of cardiomyocyte transcription factor Nkx2.5 increases 4 to 6 days after XAV939 treatment. Expression levels shown are normalized to Day 0 expression levels. DMSO+V is DMSO induction with no XAV939 after FACS. DMSO+XAV is DMSO induction plus XAV939 addition after FACS. DM+V is DM induction with no XAV939 after FACS. DM+XAV is DM induction plus XAV939 addition after FACS. DMH1+V is DMH1 induction with no XAV939 after FACS. DM+XAV is DMH1 induction plus XAV939 addition after FACS.
(TIF)

Figure S2 **Gene expression analysis of individual experiments for mesoderm and cardiogenic markers for experiment No.2.** Induction of mesoderm and cardiac specific markers in the GFP– fractions after Wnt inhibition were analyzed using rt-PCR and shown as individual experiments in each figure. XAV939 (1 µM) upregulated Mesp1 expression 2 to 4 days after treatment in the sample initially induced using DMH1. The DMH1-treated fraction shows increased Isl1 expression 4 days after XAV939 treatment (Day 8 post-DMH1 induction). The expression of cardiomyocyte transcription factor Nkx2.5 increases 4 to 6 days after XAV939 treatment. Expression levels shown are normalized to Day 0 expression levels. DMSO+V is DMSO induction with no XAV939 after FACS. DMSO+XAV is DMSO induction plus XAV939 addition after FACS. DM+V is DM induction with no XAV939 after FACS. DM+XAV is DM induction plus XAV939 addition after FACS. DMH1+V is DMH1 induction with no XAV939 after FACS. DM+XAV is DMH1 induction plus XAV939 addition after FACS.
(TIF)

Figure S3 **Gene expression analysis of individual experiments for mesoderm and cardiogenic markers for experiment No.3.** Induction of mesoderm and cardiac specific markers in the GFP– fractions after Wnt inhibition were analyzed using rt-PCR and shown as individual experiments in each figure. XAV939 (1 µM) upregulated Mesp1 expression 2 to 4 days after treatment in the sample initially induced using DMH1. The DMH1-treated fraction shows increased Isl1 expression 4 days after XAV939 treatment (Day 8 post-DMH1 induction). The expression of cardiomyocyte transcription factor Nkx2.5 increases 4 to 6 days after XAV939 treatment. Expression levels shown are normalized to Day 0 expression levels. DMSO+V is DMSO induction with no XAV939 after FACS. DMSO+XAV is DMSO induction plus XAV939 addition after FACS. DM+V is DM induction with no XAV939 after FACS. DM+XAV is DM induction plus XAV939 addition after FACS. DMH1+V is DMH1 induction with no XAV939 after FACS. DM+XAV is DMH1 induction plus XAV939 addition after FACS.
(TIF)

Figure S4 **DMH1 does not induce apoptosis 24 h after treatment.** CGR8 cells were incubated in differentiation media with or without compounds for 24 h prior to TUNEL assay. DNase-treated cells were used as positive control. Cells incubated with the labeling solution alone and without enzyme served as negative control. The results show DM can cause cellular apoptosis 24 h after treatment in one visual field, and the results are not statistically significant. No positive TUNEL staining was observed after DMH1 treatment.
(TIF)

Figure S5 **DMH1 does not induce apoptosis 48 h after treatment.** CGR8 cells were incubated in differentiation media with or without compounds for 48 h prior to TUNEL assay. DNase-treated cells were used as positive control. Cells incubated with the labeling solution alone and without enzyme served as negative control. The results show neither DM nor DMH1 causes cellular apoptosis 48 h after treatment.
(TIF)

Figure S6 **Early transient BMP inhibition is essential for cardiomyogenesis.** Gene expression profiling was performed after treatment with DM, DMH1, or DMH4. DMH4 is a VEGF-specific inhibitor with no antagonist effects on BMP signaling [21]. The data show BMP inhibition is required to upregulate cardiomyogenic gene expression, while VEGF-inhibition appeared to yield an expression profile consistent with vascular development by upregulating smooth muscle marker Myh11 and endocardial marker VE-CAM. Results are presented as the average of three independent experiments. Expression levels shown are normalized to Day 0 expression levels. Error bars denote the S.E.M. DMSO is the vehicle control. DM is dorsomorphin treatment. DMH1 is dorsomorphin homologue 1. DMH4 is dorsomorphin homologue 4.
(TIF)
