# Supplementary figures and images for: DMH1, a Novel BMP Small Molecule Inhibitor, Increases Cardiomyocyte Progenitors and Promotes Cardiac Differentiation in Mouse Embryonic Stem Cells
Source: PLoS One. 2012 Jul 27;7(7):e41627. doi: 10.1371/journal.pone.0041627 (PMC3407188; doi:10.1371/journal.pone.0041627)

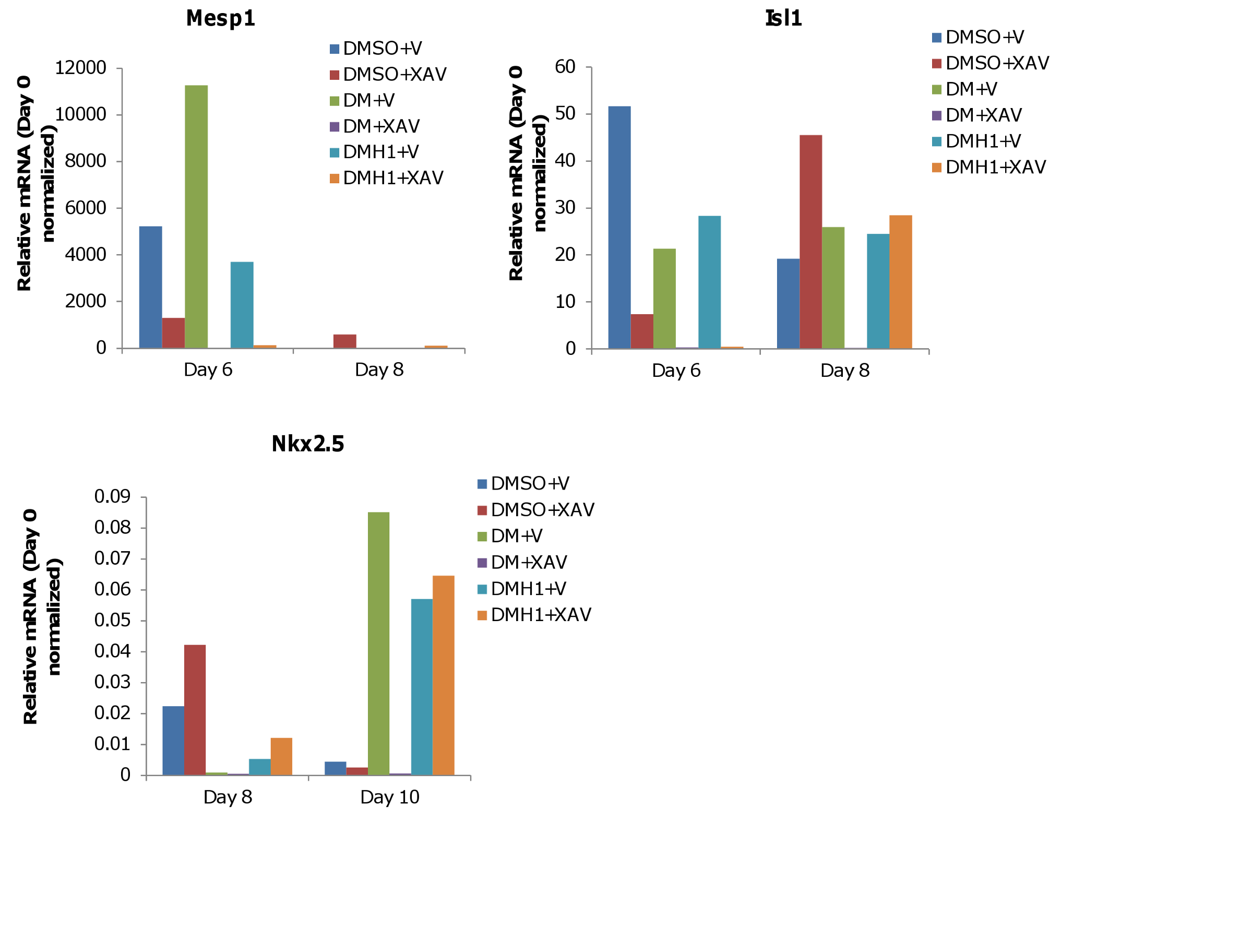

Supplement: Figure S1 — Gene expression analysis of individual experiments for mesoderm and cardiogenic markers for experiment No.1. Induction of mesoderm and cardiac specific markers in the GFP– fractions after Wnt inhibition were analyzed using rt-PCR and shown as individual experiments in each figure. XAV939 (1 µM) upregulated Mesp1 expression 2 to 4 days after treatment in the sample initially induced using DMH1. The DMH1-treated fraction shows increased Isl1 expression 4 days after XAV939 treatment (Day 8 post-DMH1 induction). The expression of cardiomyocyte transcription factor Nkx2.5 increases 4 to 6 days after XAV939 treatment. Expression levels shown are normalized to Day 0 expression levels. DMSO+V is DMSO induction with no XAV939 after FACS. DMSO+XAV is DMSO induction plus XAV939 addition after FACS. DM+V is DM induction with no XAV939 after FACS. DM+XAV is DM induction plus XAV939 addition after FACS. DMH1+V is DMH1 induction with no XAV939 after FACS. DM+XAV is DMH1 induction plus XAV939 addition after FACS. (TIF) [file pone.0041627.s001.tif]

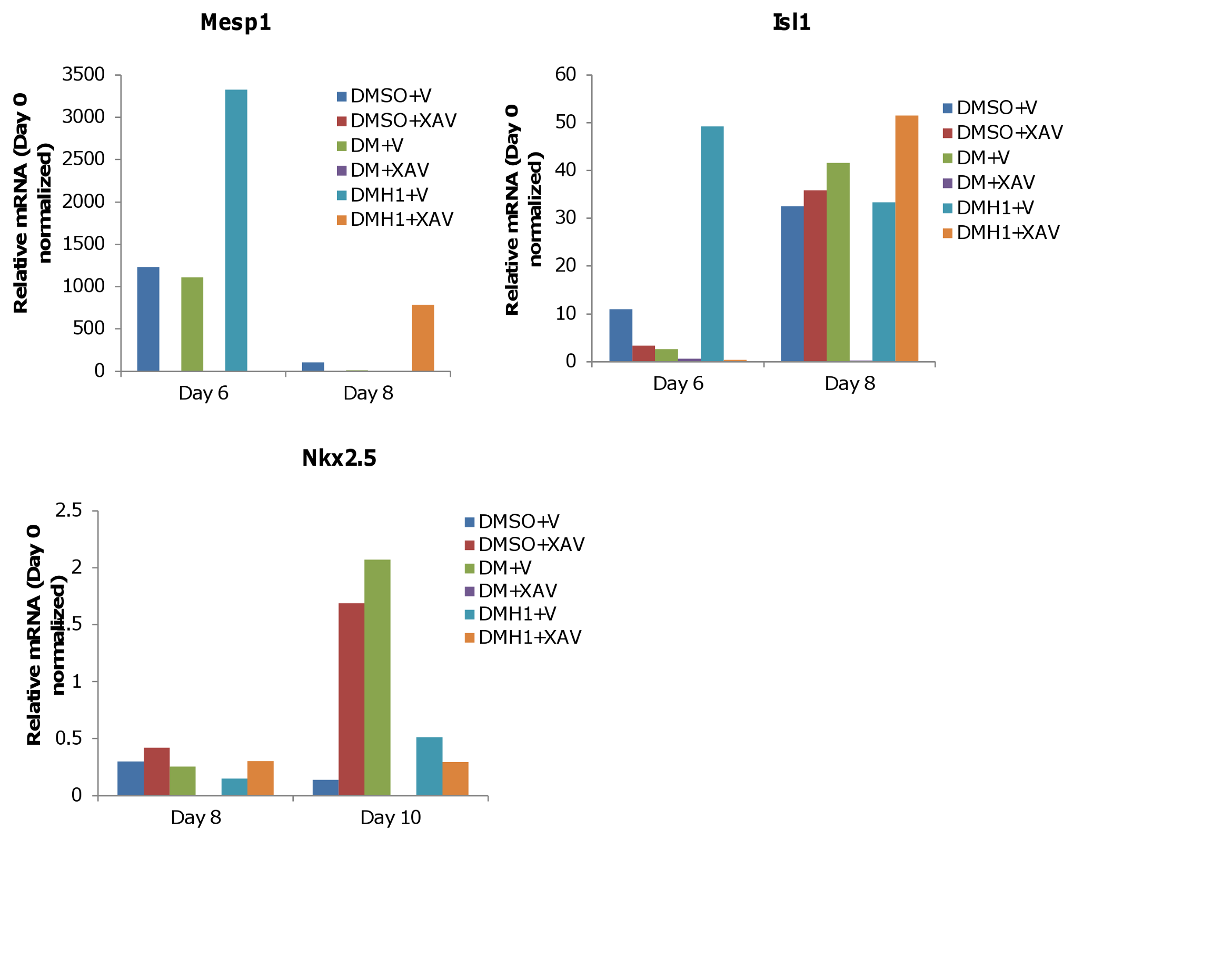

Supplement: Figure S2 — Gene expression analysis of individual experiments for mesoderm and cardiogenic markers for experiment No.2. Induction of mesoderm and cardiac specific markers in the GFP– fractions after Wnt inhibition were analyzed using rt-PCR and shown as individual experiments in each figure. XAV939 (1 µM) upregulated Mesp1 expression 2 to 4 days after treatment in the sample initially induced using DMH1. The DMH1-treated fraction shows increased Isl1 expression 4 days after XAV939 treatment (Day 8 post-DMH1 induction). The expression of cardiomyocyte transcription factor Nkx2.5 increases 4 to 6 days after XAV939 treatment. Expression levels shown are normalized to Day 0 expression levels. DMSO+V is DMSO induction with no XAV939 after FACS. DMSO+XAV is DMSO induction plus XAV939 addition after FACS. DM+V is DM induction with no XAV939 after FACS. DM+XAV is DM induction plus XAV939 addition after FACS. DMH1+V is DMH1 induction with no XAV939 after FACS. DM+XAV is DMH1 induction plus XAV939 addition after FACS. (TIF) [file pone.0041627.s002.tif]

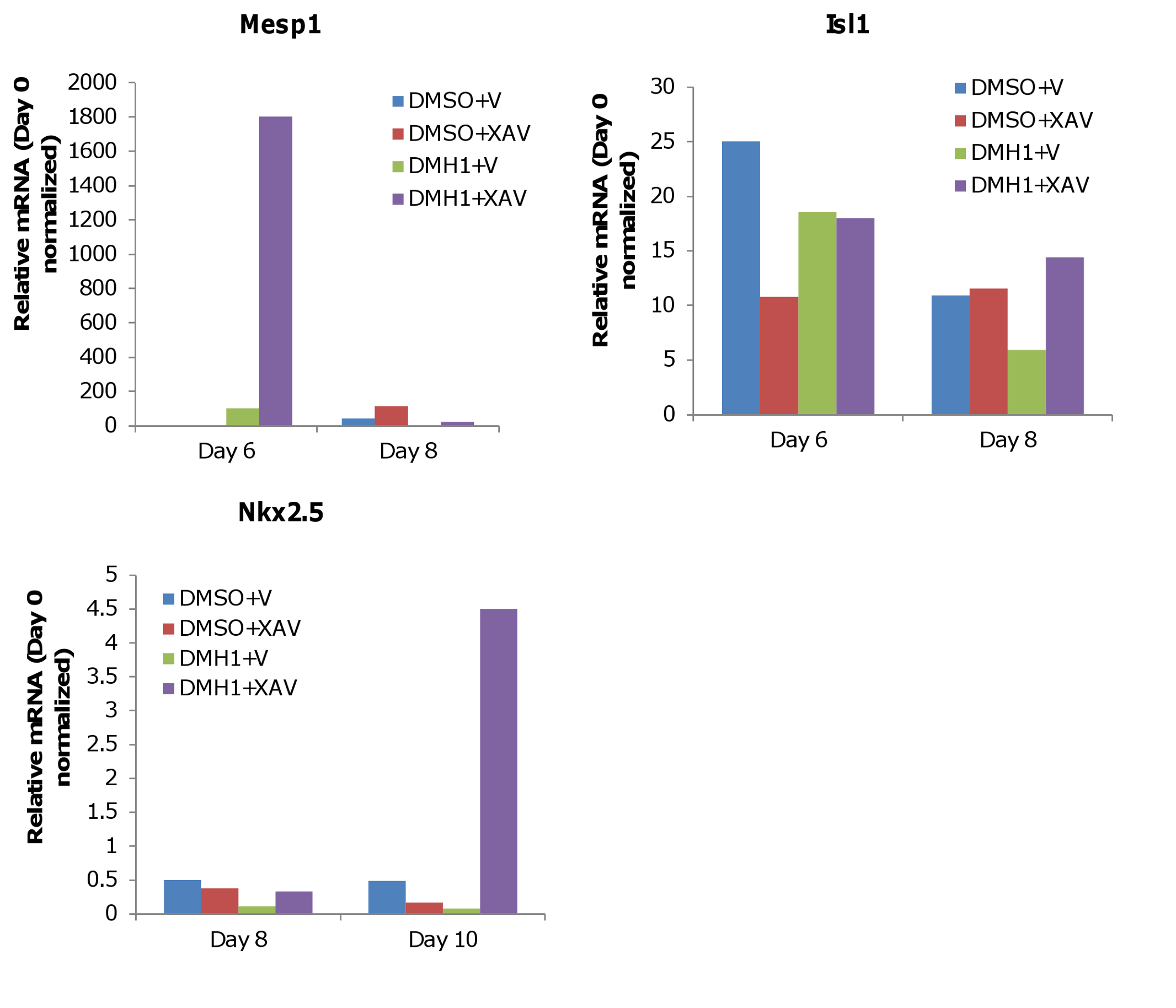

Supplement: Figure S3 — Gene expression analysis of individual experiments for mesoderm and cardiogenic markers for experiment No.3. Induction of mesoderm and cardiac specific markers in the GFP– fractions after Wnt inhibition were analyzed using rt-PCR and shown as individual experiments in each figure. XAV939 (1 µM) upregulated Mesp1 expression 2 to 4 days after treatment in the sample initially induced using DMH1. The DMH1-treated fraction shows increased Isl1 expression 4 days after XAV939 treatment (Day 8 post-DMH1 induction). The expression of cardiomyocyte transcription factor Nkx2.5 increases 4 to 6 days after XAV939 treatment. Expression levels shown are normalized to Day 0 expression levels. DMSO+V is DMSO induction with no XAV939 after FACS. DMSO+XAV is DMSO induction plus XAV939 addition after FACS. DM+V is DM induction with no XAV939 after FACS. DM+XAV is DM induction plus XAV939 addition after FACS. DMH1+V is DMH1 induction with no XAV939 after FACS. DM+XAV is DMH1 induction plus XAV939 addition after FACS. (TIF) [file pone.0041627.s003.tif]

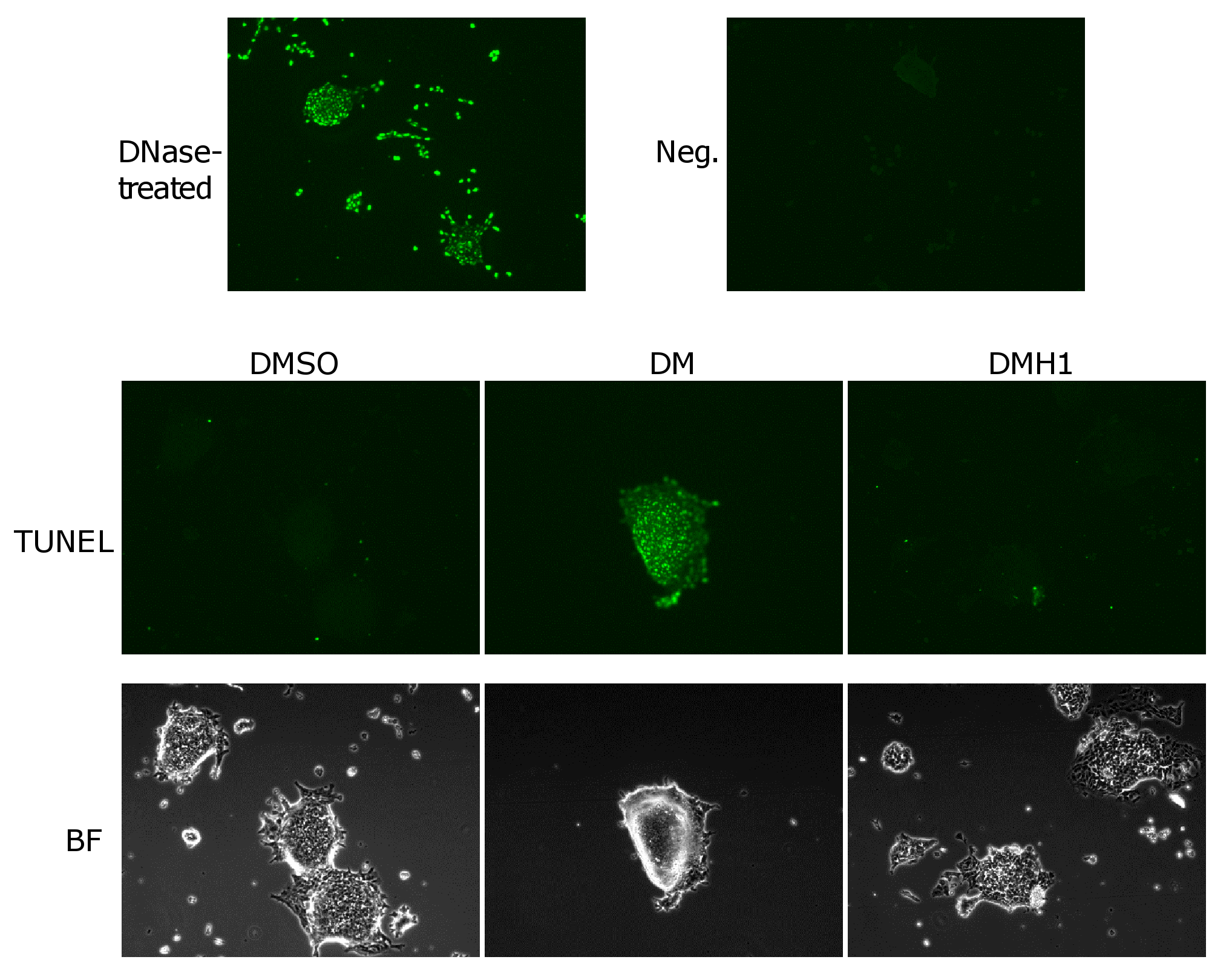

Supplement: Figure S4 — DMH1 does not induce apoptosis 24 h after treatment. CGR8 cells were incubated in differentiation media with or without compounds for 24 h prior to TUNEL assay. DNase-treated cells were used as positive control. Cells incubated with the labeling solution alone and without enzyme served as negative control. The results show DM can cause cellular apoptosis 24 h after treatment in one visual field, and the results are not statistically significant. No positive TUNEL staining was observed after DMH1 treatment. (TIF) [file pone.0041627.s004.tif]

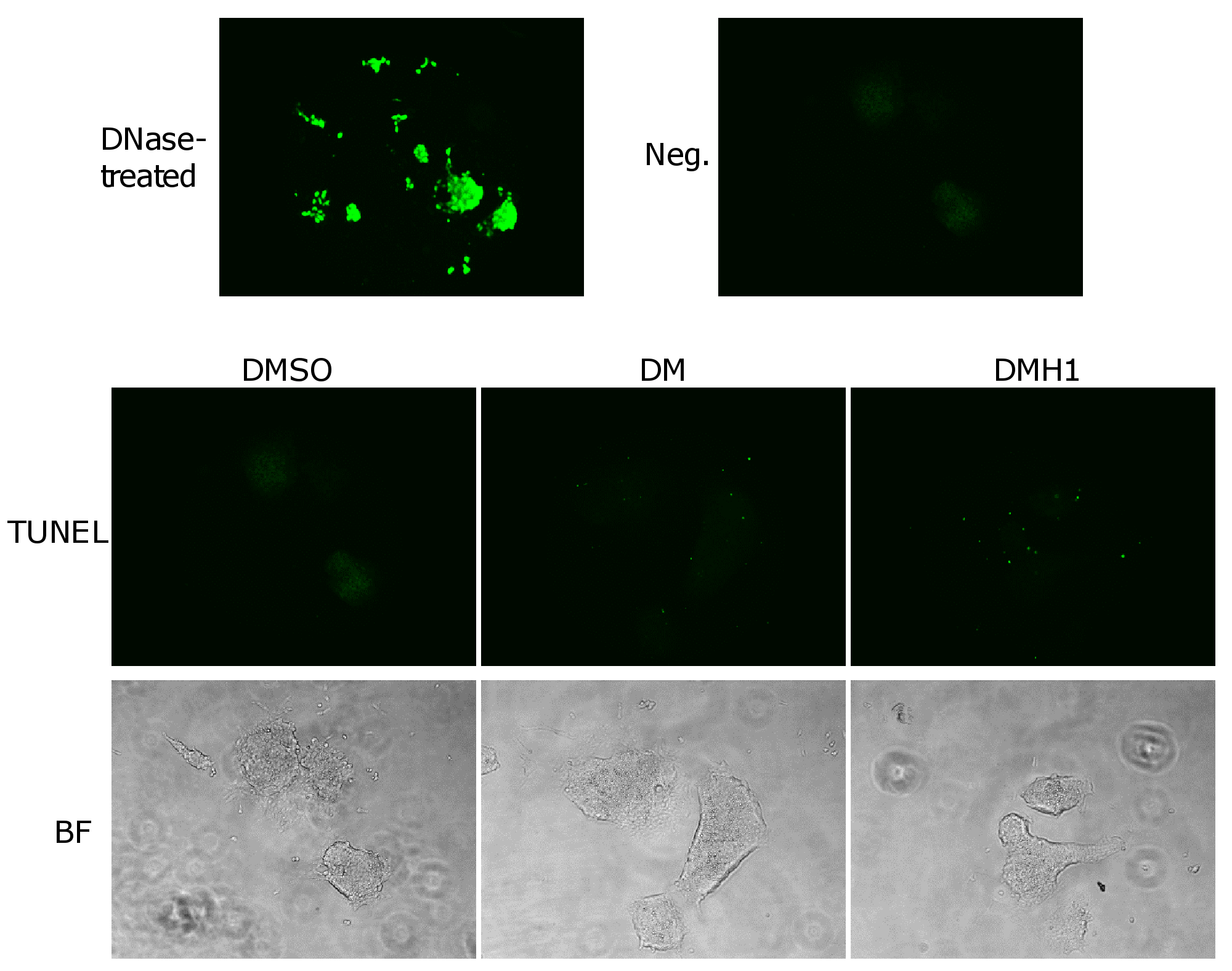

Supplement: Figure S5 — DMH1 does not induce apoptosis 48 h after treatment. CGR8 cells were incubated in differentiation media with or without compounds for 48 h prior to TUNEL assay. DNase-treated cells were used as positive control. Cells incubated with the labeling solution alone and without enzyme served as negative control. The results show neither DM nor DMH1 causes cellular apoptosis 48 h after treatment. (TIF) [file pone.0041627.s005.tif]

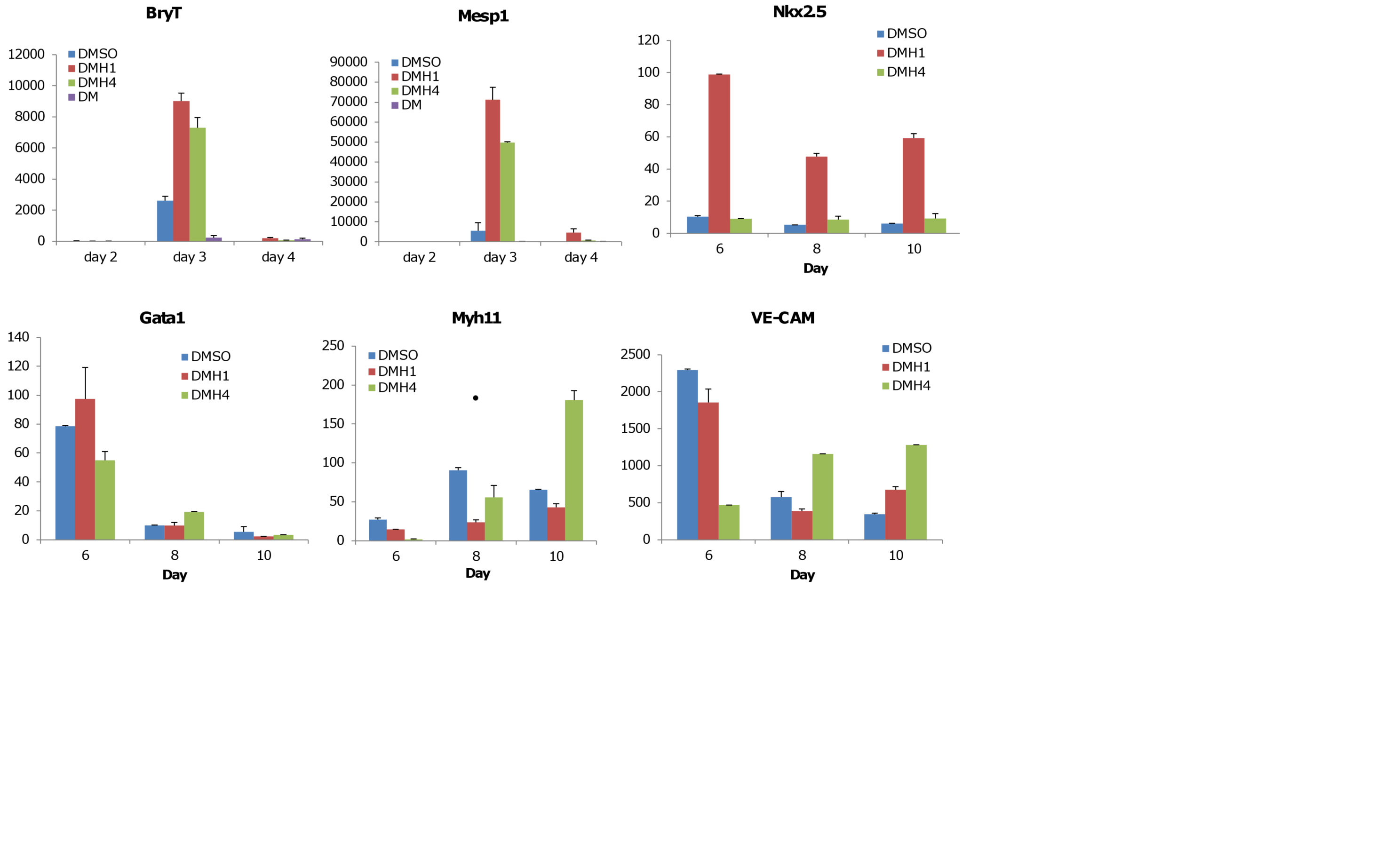

Supplement: Figure S6 — Early transient BMP inhibition is essential for cardiomyogenesis. Gene expression profiling was performed after treatment with DM, DMH1, or DMH4. DMH4 is a VEGF-specific inhibitor with no antagonist effects on BMP signaling [21]. The data show BMP inhibition is required to upregulate cardiomyogenic gene expression, while VEGF-inhibition appeared to yield an expression profile consistent with vascular development by upregulating smooth muscle marker Myh11 and endocardial marker VE-CAM. Results are presented as the average of three independent experiments. Expression levels shown are normalized to Day 0 expression levels. Error bars denote the S.E.M. DMSO is the vehicle control. DM is dorsomorphin treatment. DMH1 is dorsomorphin homologue 1. DMH4 is dorsomorphin homologue 4. (TIF) [file pone.0041627.s006.tif]
